# Understanding the importance of medical student clerkships in poor health outcome regions served by Area Health Education Centers (AHECs) in impoverished locations of Southern United States

**DOI:** 10.1186/s13690-017-0175-y

**Published:** 2017-02-13

**Authors:** Ashruta Patel

**Affiliations:** Philadelphia College of Osteopathic Medicine – Georgia Campus, 625 Old Peachtree Road NW, Suwanee, GA 30024 USA

**Keywords:** Area Health Education Centers, Southern Black Belt, Rural, Underserved, Clerkships, Rotations

## Abstract

Area Health Education Centers provide health professional students the opportunity to explore the benefits of practicing in a rural and underserved location. The status of health conditions in chronic disease patients residing in impoverished regions of the US provides the chance to understand the factors that are responsible for constant inadequate outcomes in underserved and rural communities. Many limiting barriers to positive health outcomes occur in disproportionate numbers in the Southern Black Belt. Students should consider participating in rural and underserved clerkships, and ultimately a career as a health care provider in a poor health outcome region. In addition, promising programs (e.g. telemedicine, community health workers) to help implement patient-centered evidence-based interventions can tackle current chronic disease issues commonly encountered by health professionals who work with diverse patient populations.

## Background

Through community-based training, financial assistance (housing, transportation, stipend), and partnerships throughout various counties, Area Health Education Centers (AHECs) provide valuable opportunities for all future health-professional students who are interested in pursuing careers in impoverished areas.

AHECs were created by US Congress in 1972 to address health professional shortage, increase health providers in rural and underserved communities, and create educational opportunities for students in training [1, 2]. The Health Resources and Services Administration (HRSA) financially supports national AHEC centers to help recruit, train, and retain health professionals for rural and underserved communities by providing academic and clinical resources within the community to nurture health needs [3].

Clerkships provide health professional students the chance to spotlight various aspects of rural and underserved health care careers and understand the implications of educational training in certain locations for future professional options. The implementation of health care practices in rural areas across the US is necessary to reduce emergency room visits, support appropriate health behaviors, and control chronic medical conditions before they progress in severity.

### Southern Black Belt

The term “Black Belt” is used to identify areas of the Deep South in the US because of the prevalence of its dark soil and high percentage of African American communities [4]. The Black Belt is characterized by increased rates of poverty, poor health outcomes, low literacy, heavy dependence on public assistance programs, substandard housing, high unemployment rates, poor economies, and limited access to quality health care [4, 5]. These individuals are trapped in a constant cycle of the previously mentioned factors, which in turn negatively affect health conditions. Interacting with various patients one-on-one allows successful building of relationships to better understand why a patient has a particular disease(s) and which strategies could be employed to manage, or sustain proper health conditions.

### Rural and Underserved Clerkships

Multiple areas across the United States contain medically underserved populations that possess a higher than average percentage of poor health outcomes. Many of these vulnerable communities are in rural locations, where access to crucial resources (i.e., healthy food, transportation, technology) is limited, gaps between physician and patient exist, and the frequency of chronic disease is significantly higher than in other parts of the country. Rural and underserved clerkships can provide students with an exceptional awareness of ways to influence successful health outcomes, particularly for individuals who can understand the importance of balancing literacy problems, emotional factors, physical issues, and medication adherence to influence preventive medicine in areas that need it most. The final goal of training is to essentially reduce increased costs on the current health care system and produce a network of health professionals dedicated to improving the status of chronic conditions in vulnerable communities.

### Benefits of training

Training in a rural and underserved environment is beneficial in many ways. Rural and underserved areas not only help students in training gain a solid comprehension of the factors that are responsible for poor health outcomes in susceptible populations, but also provide the opportunity to bridge the gap between healthcare professionals and ill patients. Training provides appreciation of cultural variations and perceived stigma prevalent in communities like the Southern Black Belt. Students can understand the importance of diversity and engaging in partnerships with community members to help reduce any communication barriers that might exist. Incentive programs are constantly being implemented for providers or students interested in pursuing a career as a rural or underserved primary-care physician. Successful experiences can increase the likelihood of eventually practicing in similar areas. In addition, many training sites can offer future health professionals the opportunity to incorporate new and innovative developments into practice when treating patients residing in impoverished areas.

### Future Advances

Many advances have been made in the past, and creating models of care tailored to each patient can provide promising results in isolated communities. Telemedicine employs technology, health care providers, and evidence-based models to address the barrier of geographical separation between a patient and provider in rural and underserved communities [6]. Patients residing in remote locations also have the option of using technology to connect with health care professionals. The use of interventions to reduce the rates of hospitalization is important among patients who are at high-risk of readmission into the emergency room to prevent avoidable health care costs. These approaches can be conducted through videoconferencing-based consultations during which patients interact with a health care provider and receive medical advice through video [6]. Telemedicine, therefore, not only provides appropriate advice for one’s medical condition, but also creates a successful patient-physician relationship and a strong support network. Other community-based interventions have also shown promising progresses and can be implemented in similar settings.

Community Health Workers (CHWs) can address health disparities dominant in a variety of rural and underserved locations comparable to the Southern Black Belt. CHWs serve as lay leaders in rural communities, and emphasize the importance of preventing disease and promoting health [7]. Many CHWs reside in the same community as the patients they are serving and thus further assist in closing the prevalent gap among rural populations and physicians. Nationally funded programs help finance educational partnerships and encourage students to explore careers in underserved and rural medicine.

### National Health Service Corps

The National Health Service Corps (NHSC) is well known for its efforts to reduce health care costs by increasing the prevalence of primary-care providers in rural and underserved areas across the US. The organization evaluates the conditions of locations by calculating a health professional shortage area (HPSA) score. The NHSC program participants then contribute time back into communities to help deliver quality care. Financial incentives in the form of scholarships or loan repayments help students pay for school and other relevant expenses. Implementing these programs not only offers the chance to engage in community-based experiences, but also allows future health professionals to experience and understand the factors that are causing poor health outcomes in some of the most deprived areas across the nation. These factors must be considered when designing individualized care for patients who are constantly trapped in a cycle of barriers (emotional, physical, mental) that negatively impact proper health maintenance, behaviors, and outcomes.

### Experiences and Expectations

Various factors influence medical students’ primary care or specialty career choices, including gender, race/ethnicity, socioeconomic status, previous rural or urban residence, perceptions, and interest acquired during medical school clerkships [8, 9]. Therefore, it is important to target medical students who are from certain demographics and encourage participation to practice in rural and underserved communities to aid in growing the number of health care providers practicing in vulnerable regions of the US. Furthermore, rural and underserved medical school clerkship experiences provide an outlet to work with poor health outcome patients, and potentially foster one’s interest to pursue a career caring for similar individuals.

## Conclusions

Medical students should participate in clerkship experiences funded through AHEC centers across the nation. Teaching opportunities in underserved and rural populations can help alleviate the burden of increased health care costs and poor health outcomes in susceptible regions, such as the Southern Black Belt. Increasing the number of practicing health care providers in communities affected by health disparities and social determinants of health can mitigate some of these concerns. More awareness about the benefits associated with medical student clerkship programs can promote rural and underserved community career options through a variety of means, including financial incentives funded by organizations like the NHSC. Future advancements in the disciplines of telemedicine and CHWs can further influence quality health care in impoverished communities with poor health outcomes.

## Abbreviations

AHEC: Area Health Education Center
CHW: Community Health Worker
HPSA: Health Professional Shortage Area
HRSA: Health Resources and Service Administration
NHSC: National Health Service Corps
